# Hepatotoxicity of antipsychotics: an exploratory pharmacoepidemiologic and pharmacodynamic study integrating FAERS data and *in vitro* receptor-binding affinities

**DOI:** 10.3389/fpsyt.2024.1479625

**Published:** 2024-10-14

**Authors:** René Zeiss, Carlos Schönfeldt-Lecuona, Bernhard J. Connemann, Susanne Hafner, Maximilian Gahr

**Affiliations:** ^1^ Department of Psychiatry and Psychotherapy III, University of Ulm, Ulm, Germany; ^2^ Institute of Experimental and Clinical Pharmacology, Toxicity and Pharmacology of Natural Products, University of Ulm, Ulm, Germany; ^3^ District Hospital for Psychiatry, Psychotherapy and Psychosomatic Medicine Schloss Werneck, Werneck, Germany

**Keywords:** antipsychotic agents, chemical and drug induced liver injury, receptors serotonin, pharmacovigilance, receptor binding

## Abstract

**Introduction:**

Antipsychotic psychopharmacotherapy is associated with the risk of drug-induced liver injury (DILI). However, understanding specific risk factors remains challenging due to limited data. This study investigates the relationship between receptor binding affinities and occupancies of antipsychotics and their associated hepatotoxic risks.

**Methods:**

A disproportionality analysis with calculation of the Reporting Odds Ratio (ROR) and the Information Component (IC) was conducted using data from the FDA Adverse Event Reporting System (FAERS) to identify signals related to the Standardised MedDRA Query “drug-related hepatic disorders”, which served as a proxy for drug-induced hepatotoxicity. This was followed by a pharmacoepidemiologic-pharmacodynamic approach to investigate the relationship between the ROR and substance-related receptor binding affinities and occupancy, which was estimated based on *in vitro* receptor-binding profiles.

**Results:**

Significant signals were identified for several antipsychotics, including chlorpromazine, loxapine, olanzapine, and quetiapine, with chlorpromazine and loxapine showing the highest RORs for DILI. Gender-specific analysis revealed a higher frequency of signals in female patients. Statistically significant negative correlations were identified between the ROR for drug-related hepatic disorders and the affinity for serotonin receptor 5-HT1A (r (17) = -0.68, p = 0.0012), while a positive correlation was observed for cholinergic receptors (r (17) = 0.46, p = 0.048). No significant correlations were found related to other receptors or drug properties.

**Conclusion:**

Our findings suggest that the serotonin and probably the cholinergic system may play a role in the development of DILI related to antipsychotic medications. The identification of antipsychotics with a higher association with DILI, such as chlorpromazine, underscores the need for careful monitoring in clinical practice. However, our findings need further longitudinal studies to confirm causality. A better understanding of the associations may inform clinical decision-making, particularly in patients with an increased susceptibility to liver damage.

## 1 Introduction

Drug-induced-liver-injury (DILI) is one of the major risk factors for hospitalization and liver failure (1). DILI can be classified into an intrinsic form, which is dose-dependent and usually predictable, and an idiosyncratic form, which is unpredictable and dose-independent (2). The idiosyncratic form of DILI is a common phenomenon occurring under treatment with many substances. However, it is difficult to study DILI in systematic case-control studies due to the comparatively low incidence of 1 in 2000 to 1 in 100,000 exposures (2–5). Thus, population-based studies of large adverse drug reaction (ADR) reporting systems are of particular relevance to study DILI. Pharmacovigilance studies that analyze large spontaneous reporting systems, taking into account the inherent limitations of such data, are particularly useful for investigating adverse drug reactions (ADRs) that occur infrequently. This is because they consider large data sets drawn from real-world data (6). Known risk factors for the occurrence of DILI include high daily recommended doses, formation of reactive metabolites, mitochondrial toxicity, and induction of oxidative stress (7). Some parameters, such as lipophilicity, which in addition to high daily dosing is part of the so-called “rule of two” that has been proposed as a screening tool for substances with a high risk of DILI, may also play a role (8). Some studies show an association with molecular weight, although the evidence is still unclear (9, 10). Additionally, patient-specific factors such as older age, polypharmacy, and genetic variants can increase the risk of DILI (7). Polypharmacy, high daily dosages and the necessity of prolonged intake, all factors that can favor DILI, are typical features in the treatment of psychiatric disorders (11, 12). Especially the treatment of patients with schizophrenia often involves lifelong therapy and polypharmacy (13–16). In addition, the treatment is complicated by often poorer general medical care and the occurrence of various somatic comorbidities and side effects, in particular metabolic disorders, which can have among others a negative effect on liver function (17–19). Therefore, understanding the risk factors for DILI is particularly important in the treatment of schizophrenia, but also other disorders that might be treated with antipsychotics. Unfortunately, the currently available data regarding aetiology and pathophysiology of DILI related to antipsychotics are insufficient and difficult to explore. In general, studying antipsychotics is challenging due to the heterogeneity of these drugs, which act on various monoaminergic receptors, particularly affecting the dopaminergic, serotonergic, histaminergic, and cholinergic systems (20). In a previous work, we investigated the hepatotoxic potential of antipsychotics in an exploratory approach in a case-non-case study with data of VigiBase™ which is the world’s largest database of spontaneous reports of ADR. In the mentioned study, a signal for drug-related hepatic disorders was found for more than half of the investigated substances (21). Our previous research prompted us to perform a further analysis with the objective to identify potential risk factors for DILI associated with antipsychotics through a hypothesis-generating approach using data from the FDA Adverse Event Reporting System (FAERS). Recently, a number of studies have combined the pharmacodynamic properties of drugs with real-world pharmacoepidemiological data in order to investigate the mechanisms of antipsychotic-induced side effects: For example the role of dopamine receptors in movement disorders and the role of serotonin and histamine in diabetes (22, 23). To the best of our knowledge, there are no corresponding studies concerning the hepatotoxic potential of antipsychotics, thus, the objective of this exploratory, hypothesis-generating study is to investigate whether drug specific properties like the MW or the lipophilicity or the pharmacodynamic properties, specifically the receptor binding profiles of antipsychotics are associated with the substance-related risk of hepatotoxicity. To investigate the influence of receptor binding affinity and occupancy on the hepatotoxicity of antipsychotics, we used a pharmacoepidemiological-pharmacodynamic approach. To date, there is little literature suggesting that pharmacodynamic properties in terms of binding affinity of monoamines may play a role in hepatotoxicity. However, some studies have identified monoamine receptors, especially serotonin as regulatory components for hepatic stellate cells (24, 25). To investigate the mentioned issues, we performed a disproportionality analysis with subgroup analyses for women, men, and patients over 65 years of age and calculated correlations with receptor affinities, occupancies, and various relevant parameters such as molecular weight and the logarithm of the partition coefficient (LogP) as a measure of the lipophilicity for the results found.

## 2 Methods

### 2.1 Database

We obtained the data from FAERS. FAERS is a database that contains reports of adverse events, medication errors, and product quality complaints resulting in adverse events that were submitted to the FDA (26).To access the database the pharmacovigilance data analysis tool OpenVigil 2.1 was used (27). Open Vigil is a data analysis tool to analyses pharmacovigilance data from the FAERS database using cleaned FDA data with verified and normalized drug names (non-ambiguous names are not integrated for example) as well as options to remove duplicates. In addition, OpenVigil allows for subgroup analysis by sex or age, which was important to our research question and the tool has been supported by numerous studies of FAERS data in recent years (28–30). The search period covered the period in which the data was available in OpenVigil 2.1. (FDA data from Q4/2003-Q1/2024). The query date was June 15-18, 2024.

The codes of the Anatomical Therapeutic Chemical Classification System (ATC) were used for the search of included substances. The Medical Dictionary for Regulatory Activities (MedDRA) terminology was used for the identification of adverse drug reactions (ADR) (MedDRA version 24.0).

### 2.2 Antipsychotics studied

The antipsychotics studied were a selection of commonly used substances with long term data that are FDA-approved, as the FAERS database mainly contains reports from the United States. The substances included were aripiprazole, asenapine, brexpiprazole, cariprazine, chlorpromazine, clozapine, fluphenazine, haloperidol, iloperidone, loxapine, lurasidone, olanzapine, paliperidone, perphenazine, pimozide, quetiapine, risperidone, thiothixene and ziprasidone.

### 2.3 Definition of DILI

A case of DILI was defined as cases in the database in which a drug was primarily or secondary suspected for causing an ADR included in the standardized queries (SMQ): ‘drug-related hepatic disorders - comprehensive search’ (DRHD-CS). A SMQ is a pre-defined set of MedDRA terms in this case for “Drug-related hepatic disorders” used to identify and analyze data related to liver-related adverse events. They are validated, pre-determined sets of MedDRA terms intended to support safety analysis and reporting in pharmacovigilance (31).

### 2.4 Disproportionality analysis and definition of a signal

A signal of disproportionate reporting was defined as a lower 95% confidence interval (CI) of the ROR > 1 and a lower 95% CI of the information component (IC) > 0 (IC_025_) (6, 32, 33). The Reporting Odds Ratio (ROR) and its 95% CI were calculated as a measure of disproportionality. The ROR is a commonly used method to identify signals in spontaneous reporting databases (34). The ROR has a similarity to the odds ratios in case-control studies. In the present study cases were defined as all reports of DILI, represented by SMQ DRHD-CS. The “controls” were defined as all other cases in the database (“non-cases”). To enhance the robustness of a signal, the IC and their 95 CI was also calculated. The IC is a statistical measure employed to identify signals of disproportionate reporting within pharmacovigilance data sets. It quantifies the degree of association between a drug and an adverse event by comparing the observed and expected frequencies of reports (32). Only substances with at least three cases were included in the analysis. To increase the specificity of our analysis of DILI, we included only reports with the role of drug characterized as “primary or secondary suspect”. Reports with a level of “interacting” or “concomitant” were excluded. For data cleansing and deduplication, the cleaned version of OpenVigil 2.1 and only uniquely identifiable case IDs with no overlapping in gender and age and reported ADR were used. To further analyze the influence of gender and age, a subgroup analysis was performed for women, men, and patients over 65 years of age in accordance with the recommendations of the Good Signal Detection Practices (IMI PROTECT) (35).

### 2.5 Data on receptor affinity and occupancy

The following receptors were considered: dopamine receptors (D2, D3), serotonin receptors (5-HT1A, 5-HT2A, 5-HT2C and 5-HT7), histamine receptor (H1), adrenergic receptors (α1-adrenergic, α2-adrenergic, irrespective of the specific subtype), and cholinergic receptors (irrespective of the specific subtype). Receptor occupancy was calculated according to the receptor occupancy theory (36). Occupancy (%) is often expressed as 100*(CU/(Ki + CU) In which Ki is the inhibitory constant and CU the unbound drug concentration in blood in nM. CU is calculated as CU = 1000* FU *CT/MW. In this formula FU is the unbound drug fraction, CT is the blood drug concentration in ng/ml, and MW represents the molecular weight. The *in vitro* Ki values (nM) for human receptors were sourced from a previously published study by Cepaityte and colleagues (29). In their study, Cepaityte and colleagues obtained the Ki values through a number of sources: The primary source for the *in vitro* Ki values for human receptors was the Psychoactive Drug Screening Program (PDSP) database (37). When Ki values could not be obtained from the PDSP, additional data were obtained from the IUPHAR/British Pharmacological Society database (38). The International Union of Basic and Clinical Pharmacology (IUPHAR)/British Pharmacological Society (BPS) Guide to PHARMACOLOGY is an expert-curated database of ligand-activity-target relationships. In instances where multiple Ki values were available for receptors, the median value was calculated by Cepaityte et al. to ensure robust representation of binding affinity. The MW of antipsychotics was obtained from the International Union of Basic and Clinical Pharmacology (IUPHAR) database, while the unbound drug fraction (FU) was sourced from DrugBank, respectively the study by Cepaityte et al. when not available (29, 38, 39). In order to estimate the CT (total drug concentration in blood), the upper limit of the therapeutic reference range was employed (40). Receptor occupancy is a commonly used method to approximate the activity of antipsychotics at drug receptors. However, there is a risk of missing data because the calculation requires a large amount of data, some of which is based on assumptions or estimates (41). Therefore, we performed an analysis using pKi values and an additional analysis using occupancy to reduce the proportion of missing data and the risk of missing a result and for sensitivity analysis. An overview of the pKi (S4) and the calculated values for occupancy (S5) can be found in the supplements.

### 2.6 FDA approval years and partition coefficient

The FDA approval years were obtained directly from the official FDA homepage (42). The LogP values were extracted from DrugBank; LogP, or the partition coefficient, is a measure of a compound’s lipophilicity (39). An overview of the values can be found in the supplements (S6)

### 2.7 Statistical analysis

Pearson correlation coefficients were calculated to assess the relationship between pharmacodynamic and pharmacokinetic data and ROR values. Statistical significance was determined using a threshold of p < 0.05. In consideration of the hypothesis-generating character of our study, no correction for multiple testing was applied. Scatter plots with regression lines were generated to visualize the relationship between receptor affinities and ROR values. Data analysis was performed with “R” (version 4.0.0), a free software environment for statistical computing and graphics (https://www.r-project.org).

## 3 Results

### 3.1 Disproportionality analysis

At the time of the query (period Q4/2003-Q1/2024), the adjusted FAERS database included 12,345,128 cases, of which 364,647 were associated with the SMQ “drug related hepatic disorders - comprehensive search”. An overview of the number of cases for each substance, disaggregated by gender, can be found in the supplementary materials, **Supplementary Table S1**. Four signals were identified through the disproportionality analysis conducted on the entire data set under consideration: Loxapine ROR: 2.74 [2.38, 3.16], IC: 1.38 [1.00, 1.75]; chlorpromazine ROR: 2.59 [2.29, 2.93], IC: 1.30 [0.98, 1.62]; olanzapine ROR: 1.76 [1.69, 1.83], IC: 0.78 [0.68, 0.87], and quetiapine ROR: 1.13 [1.09, 1.17], IC: 0.17 [0.09, 0.25]. If only the cases related to female patients were considered, five signals were identified, encompassing the four aforementioned signals for loxapine ROR: 3.16 [2.54, 3.93], IC: 1.56 [0.96, 2.16]; chlorpromazine ROR: 2.92 [2.43, 3.52], IC: 1.46 [0.97, 1.96]; olanzapine ROR: 1.96 [1.84, 2.08], IC: 0.93 [0.78, 1.07]; quetiapine ROR: 1.30 [1.24, 1.37], IC: 0.36 [0.26, 0.47] and an additional signal for risperidone with a ROR: 1.50 [1.39, 1.62], IC: 0.56 [0.39, 0.74]. Three signals were identified for the male subgroup, specifically for loxapine ROR: 2.26 [1.84, 2.77], IC: 1.10 [0.60, 1.60]; chlorpromazine ROR: 2.22 [1.85, 2.66], IC: 1.08 [0.64, 1.53]; and olanzapine ROR: 1.60 [1.51, 1.70], IC: 0.64 [0.52, 0.77]. Four signals were identified for the category comprising individuals aged 65 years and above: chlorpromazine ROR: 3.45 [2.63, 4.53], IC: 1.64 [0.89, 2.38]; loxapine ROR: 2.58 [1.68, 3.96], IC: 1.24 [0.17, 2.31]; olanzapine ROR: 1.54 [1.37, 1.73], IC: 0.59 [0.34, 0.85] and risperidone ROR: 1.25 [1.10, 1.43], IC: 0.31 [0.04, 0.58]. A summary of the ROR for the four categories and their 95% CI is provided in **Table 1**. The calculated ICs and their 95% CI can be found in the **Supplementary**-**Table S2**.

**Table 1:** Reporting odds ratios and the respective 95% confidence interval for drug related hepatic disorders - comprehensive search (SMQ) and the categories “Total”, “Female”, “Male”, and “Age ≥ 65 years “.

Substance	Total	Female	Male	Age ≥ 65 y
	ROR (95% CI)	ROR (95% CI)	ROR (95% CI)	ROR (95% CI)
Aripiprazole	0.76 (0.72-0.80)	0.75 (0.69-0.81)	0.84 (0.78-0.90)	0.98 (0.82-1.17)
Asenapine	0.33 (0.26-0.42)	0.33 (0.23-0.47)	0.36 (0.24-0.53)	NA
Brexpiprazole	0.20 (0.15-0.26)	0.21 (0.15-0.30)	0.28 (0.19-0.42)	0.32 (0.15-0.68)
Cariprazine	0.33 (0.25-0.45)	0.31 (0.19-0.50)	0.29 (0.16-0.51)	NA
Chlorpromazine	**2.59 (2.29-2.93)**	**2.92 (2.43-3.52)**	**2.22 (1.85-2.66)**	**3.45 (2.63-4.53)**
Clozapine	0.77 (0.74-0.81)	0.89 (0.82-0.96)	0.74 (0.70-0.78)	0.39 (0.33-0.46)
Fluphenazine	**1.63 (1.20-2.21)**	**2.12 (1.30-3.46)**	1.19 (0.77-1.84)	NA
Haloperidol	1.02 (0.93-1.12)	1.08 (0.93-1.26)	0.94 (0.83-1.07)	1.10 (0.89-1.38)
Iloperidone	0.17 (0.07-0.42)	0.44 (0.18-1.06)	NA	NA
Loxapine	**2.74 (2.38-3.16)**	**3.16 (2.54-3.93)**	**2.26 (1.84-2.77)**	**2.58 (1.68-3.96)**
Lurasidone	0.27 (0.23-0.32)	0.24 (0.18-0.31)	0.30 (0.22-0.42)	0.16 (0.05-0.48)
Olanzapine	**1.76 (1.69-1.83)**	**1.96 (1.84-2.08)**	**1.60 (1.51-1.70)**	**1.54 (1.37-1.73)**
Paliperidone	0.29 (0.26-0.33)	0.47 (0.38-0.58)	0.27 (0.23-0.31)	0.35 (0.17-0.73)
Perphenazine	1.24 (0.88-1.74)	**1.92 (1.26-2.92)**	0.54 (0.26-1.14)	0.90 (0.29-2.85)
Pimozide	1.14 (0.63-2.08)	1.59 (0.65-3.89)	1.11 (0.46-2.72)	NA
Quetiapine	**1.13 (1.09-1.17)**	**1.30 (1.24-1.37)**	0.93 (0.87-0.99)	**1.13 (1.03-1.25)**
Risperidone	0.82 (0.79-0.86)	**1.50 (1.39-1.62)**	0.59 (0.55-0.63)	**1.25 (1.10-1.43)**
Thiothixene	0.97 (0.48-1.97)	1.01 (0.37-2.73)	0.96 (0.30-3.04)	NA
Ziprasidone	0.51 (0.44-0.60)	0.53 (0.42-0.66)	0.50 (0.39-0.64)	0.43 (0.20-0.91)

Signals identified are presented in bold font. A signal is defined as a lower 95% confidence interval greater than 1. NA, no data available or number of individual case safety reports is <3. ROR, Reporting Odds Ratio. CI, Confidence Interval.

### 3.2 Pharmacodynamic analysis

**Figure 1:**
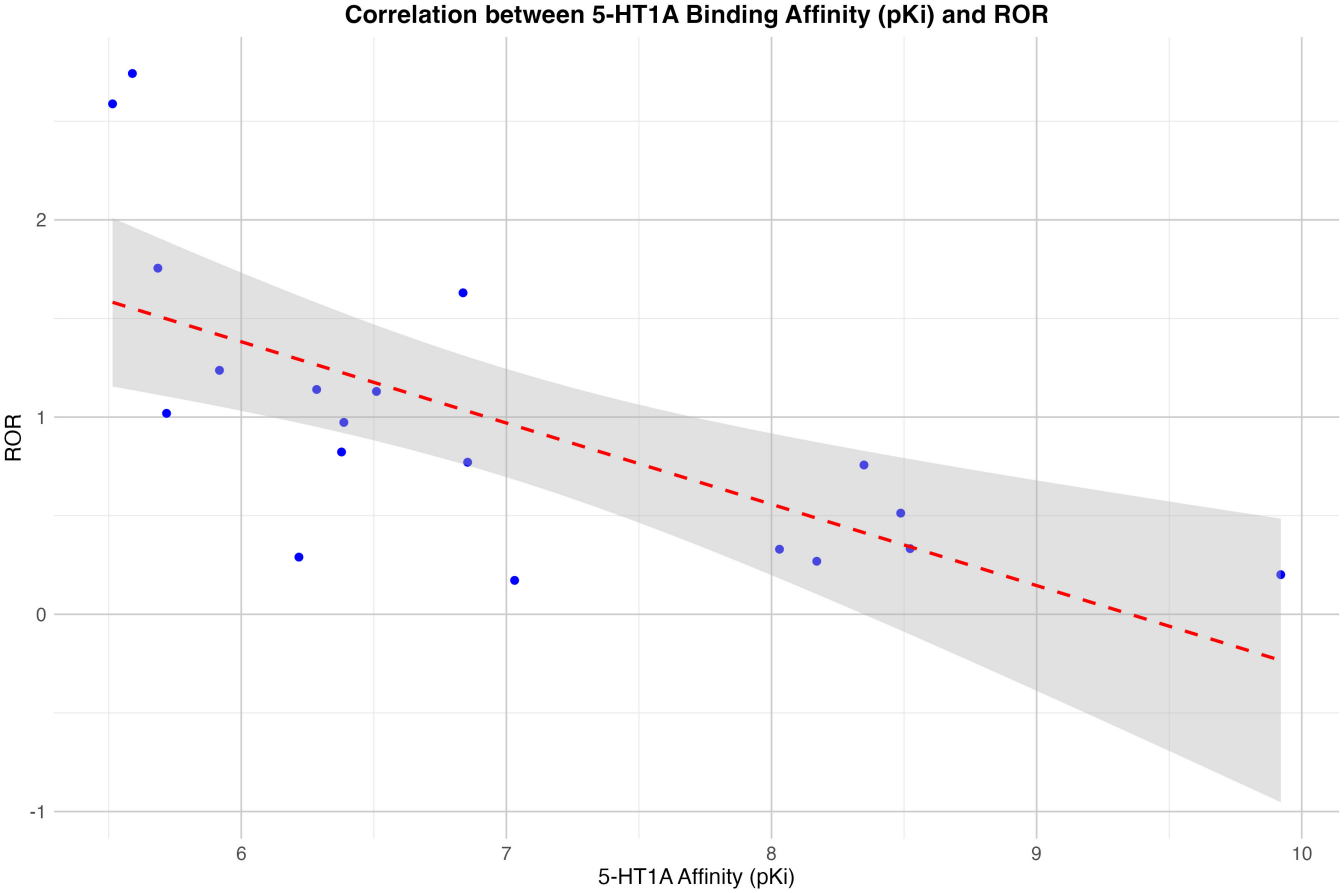
Correlation between the receptor binding affinity for the serotonin 5-HT1A-receptor (in pKi) and the Reporting Odds Ratio (ROR). The linear regression line is shown in red, the 95% confidence interval in grey.

The analysis revealed a statistically significant negative correlation (r (17) = -0. 68, p = 0.0012) between 5-HT1A affinity and drug-related liver injury. Additionally, a negative correlation was observed for occupancy of 5-HT1A, although this was slightly above the significance level: (r (17 = -0.45, p = 0.054). For the cholinergic receptors a positive significant correlation was found between the pKi and the ROR: r (17) =0.46, p = 0.048. There was no significant correlation between the ROR and the occupancy for cholinergic receptors. For the other receptors studied, there was no correlation between substance-related RORs for the SMQ ‘drug-related hepatic disorders - comprehensive search’ and receptor-specific affinities or occupancies: dopamine receptors (D2, D3), serotonin receptors (5-HT2A, 5-HT2C and 5-HT7), histamine receptor (H1), adrenergic receptors (α1-adrenergic, α2-adrenergic. The gender specific subgroup analysis showed for males significant findings for the same receptors, for females the positive correlation for the cholingergic receptor was not significant (p=0.09), however the negative correlation for the 5-HT1A occupancy was significant in the female group with a p-value of 0.02. A scatter plot with the findings for the affinity of 5-HT1A for the entire dataset is shown in **Figure 1**.

### 3.3 Analysis of other parameters

The further analysis showed a negative significant correlation between the ROR and the MW: r (17) = -.47, p = 0.04 and a negative significant correlation between the ROR and the year of approval: r (17) = -.5, p = 0.029. The findings remained significant in the gender specific subgroup analysis for the year of approval, however the correlation between the ROR and the MW was not significant in the female subgroup. The LogP showed no significant correlations with the ROR. A summary of the results of the correlations can be found in the **Supplementary Materials (Supplementary**-**Table S3**).

## 4 Discussion

### 4.1 Signals of disproportionate reporting

We found several signals of disproportionate reporting for antipsychotics and hepatotoxicity, namely for chlorpromazine, loxapine, olanzapine and quetiapine. Overall, the ROR and IC values were relatively low, which fits with the general observation that antipsychotics are not associated with a very high hepatotoxic potential, however the identified signals fit well in the available literature (43). Cases of liver damage have been described for chlorpromazine since the 1950s and it is considered one of the antipsychotics with the highest hepatotoxic potential (44, 45). Of the atypical antipsychotics, clozapine, olanzapine, quetiapine and risperidone are frequently associated with hepatotoxicity, in particular serum enzyme elevations (43). Accordingly, a signal was found in our analysis for olanzapine and quetiapine and for risperidone in the female subgroup. We were unable to find a signal for clozapine, but this may also be related to the strict laboratory controls due to possible agranulocytosis and therefore an early stop of treatment if there are signs of hepatotoxicity and because of the comparatively rare use, despite its effectiveness, due to the complex side effect profile (46–48). It is noteworthy that the subgroup analysis of female patients indicated a greater number of signals than the overall dataset or the analysis of male patients, which is consistent with the existing literature indicating that gender may be a potential risk factor for DILI (49, 50). Gender differences in liver enzyme activity may impact drug pharmacokinetics and pharmacodynamics (51). The analysis of the patient group aged 65 and over revealed greater difficulties in implementation due to the lower number of cases. However, some signals were identified that may provide a basis for suggesting a link between the risk of DILI and the use of antipsychotics in this age group. Age can affect liver function and the body’s ability to process medications, with older adults often experiencing slower metabolism and increased risk of DILI (52). The signals found are of relevance for everyday clinical practice if they are confirmed in further studies, since olanzapine and especially quetiapine are also increasingly used outside the treatment of schizophrenia (53). Quetiapine for example is increasingly used in sleep-inducing indications (54, 55). However, the substance has a broad spectrum of receptor activity extending beyond the sleep-inducing antihistaminergic effect, and the signal found here suggests that liver function tests should be checked regularly when quetiapine is used, especially in older patients and women.

### 4.2 Pharmacodynamic analysis

Furthermore, the results of our analysis indicated a significant negative correlation between the ROR for DILI respectively for the SMQ ‘drug-related hepatic disorders - comprehensive search’ and the affinity for the serotonin receptors 5-HT1A as well as a trend for the receptor occupancy for 5-HT1A. It is crucial to emphasize that ROR is not conceptualized as a relative risk, and that a significant correlation with receptor affinity does not constitute causal evidence. The existing literature on the relationship between the extent of disproportionality and potential risk is controversial.Some literature suggest that disproportionality analyses in spontaneous reporting databases may correlate with the risk of an event, particularly the degree of disproportionality (35). A recent study investigating the relationship between risks from meta-analyses and data from pharmacovigilance disproportionality analyses concluded that although they often correlate, it is highly dependent on the type of response. More objectifiable ADRs appear to show a greater correlation (56). The data presented here suggest that increased affinity to the 5-HT1A receptor may confer a decreased risk for hepatotoxic effects.

To the best of our knowledge, this is the first analysis of data of a spontaneous reporting database in regards of the association between receptor binding profiles of antipsychotics and hepatotoxicity. Our analyses suggest that the serotonin system is involved. This is in line with previous studies in animal models: Rudell et al. demonstrated that both rat and human hepatic stellate cells (HSC) express multiple serotonin receptors, including 5-HT1B, 5-HT1F, 5-HT2A, 5-HT2B, and 5-HT7. In their study, they found that the inhibition of 5-HT2 receptors (specifically 5-HT2A and 5-HT2B) reduces the proliferation of HSCs and increases apoptosis, indicating a protective role against fibrotic activity. Furthermore, 5-HT2B receptor expression is associated with fibrotic tissues, and its antagonism could help mitigate fibrosis (24). A study by Ebrahimkhani et al. provided evidence that the 5-HT2B receptor plays a critical role in liver fibrosis and regeneration. Their findings suggest that antagonism of 5-HT2B enhances hepatocyte proliferation and reduces fibrogenesis) (25). Insufficient data and limited affinity for the 5-HT2B receptor in our study preclude precise statements on the 5-HT2B. However, the studies referenced are in alignment with our findings, as they similarly indicate a role for serotonin receptors in liver repair mechanism and DILI. One of the proposed mechanisms is the reduction in TGF-β1 expression resulting from 5-HT2B antagonism. The results of a recent study were consistent with those discussed above: In their 3D human liver spheroid model, the authors demonstrated that antagonists of 5-HT receptors, including ketanserin and sarpogrelate, significantly reduced free fatty acids-induced fibrosis by decreasing the expression of key fibrotic markers such as COL1A1, TGF-β1, and vimentin (57). While the antagonism via ketanserin at 5HT2A was most significant in their work, the results still support our findings that an antagonistic effect on serotonin receptors may play a role in DILI. The exact mechanism by which antagonism of serotonin receptors, particularly 5-HT2A and 5-HT2B, might protect against liver fibrosis remains unclear. The aforementioned studies indicate that the effect of serotonin receptor antagonists on TGF-β1 signaling pathways and induction of apoptosis in hepatic stellate cells (HSCs) may be a contributing factor.

Furthermore, our analysis showed a positive correlation between substance affinity to cholinergic receptors and the ROR for DILI. There is little evidence on the role of cholinergic receptors in DILI in humans. However, in an mouse models of azoxymethane-induced liver injury, M1 receptor deficiency reduced hepatocyte apoptosis and diminished liver fibrosis by anti-oxidant effects indicating that the M1 receptor modulates acute and chronic liver injury (58, 59). Based on these findings in animals and the result of our study, one might assume that M1 receptor affinity interferes with anti-oxidant responses in hepatocytes. The various subtypes of cholinergic receptors appear to exert distinct effects on the liver (60). However, given the non-selective anticholinergic effect of the investigated substances in our study, no precise statement can be made regarding this aspect based on our available data. Further studies could investigate the role of cholinergic receptors by including a large number of anticholinergic substances that may act specifically on individual subtypes of the receptor. A further aspect that has to be considered is that the correlation between cholinergic affinity and drug-related liver injury might be explained by the further development of antipsychotic therapies and improvements in terms of adverse effects of all kind in more recent years. Nowadays, drug development includes the monitoring of adverse substance effects from the very beginning and candidates with a higher potential of side effects are less likely to be further developed. Anti-cholinergic effects of medications are associated with a higher risk of side effects, including dry mouth, urinary retention, constipation, increased heart rate and visual disturbances. Thus, the correlation between cholinergic affinity and risk of DILI might be associated with the avoidance of anti-cholinergic drug candidates in more recent years. This assumption is consistent with our results in that the year of approval correlated negatively with the ROR. Nonetheless, the clinical relevance of substance affinity to cholinergic receptors and the risk of DILI needs to be addressed by future studies. In addition, other receptors as the dopamine receptors were considered. However, no statistically significant correlation was found between the affinity or occupancy for these receptors and the ROR for DILI. In summary, the results of our study are in line with the result from previous animal studies that highlight the involvement of serotonin and cholinergic systems in liver injury and repair mechanisms. Specifically, our observation of a negative correlation between 5-HT1A receptor affinity and DILI is generally consistent with animal models showing that inhibition of serotonin receptors, particularly 5-HT2A and 5-HT2B, plays a protective role in liver fibrosis. Although our study did not directly assess 5-HT2B receptor affinity, the mechanistic insights from animal models suggest that serotonin receptor antagonism, particularly 5-HT2B, could contribute to decreased liver fibrosis and injury, supporting our findings on a rather protective role of the serotonergic system in liver repair mechanisms.

Additionally, the study indicated a negative correlation between molecular weight and DILIs. It is worth noting that this particular finding was not observed in the gender-specific analysis of female subjects. In the available literature, a higher molecular weight appears to be more of a risk factor for DILI. Nevertheless, this is more apparent for substances with a MW greater than 600 g/mol, and none of the substances under examination fell within this range (9). The results obtained regarding a potential correlation between the receptor profile and reports of DILI do not yet allow any definitive conclusions to be drawn for clinical practice. Nevertheless, the results suggest that there may be a potential correlation between receptor activity on the monoamine system and an impact on hepatic metabolism or the risk of hepatotoxicity. This should be considered in future studies and in the development of new substances.

### 4.3 Limitations

Our study also contains some limitations. One limitation is due to the nature of the study. Despite careful evaluation and control for duplicates, the information in the FAERS-database originates from a variety of sources. Consequently, the probability that a suspected adverse effect is drug-related cannot be assumed to be the same in all cases. In order to mitigate the impact of this issue, our approach was to restrict the scope of our analysis to reports in which the substance in question was also identified as a suspected substance. Another limitation is the issue of underreporting in spontaneous reporting databases, which likely leads to the underreporting of minor DILI cases (61). Underreporting is a major problem for spontaneous reporting systems in general. A major reason for underreporting is insufficient time or the low level of awareness among healthcare professionals (61–63). To mitigate this issue, a study by Shchory et al. has shown that targeted interventions such as educational programs, reminders, and simplifying the reporting process can greatly increase reporting rates (64). However, we assume that for our present work, the issue of underreporting should affect all substances equally and therefore only has a minimal impact on the main results of our study.

Another limitation affects the data regarding receptor affinity. Variability in measurement methods across different studies, including differences in assay types and experimental conditions, can lead to inconsistencies in reported affinity values. As noted by Landrum and Riniker, the integration of Ki data from different sources inevitably introduces considerable noise, which can be attributed to a number of factors, including differences in experimental conditions such as buffer composition, temperature, and duration, as well as technological differences in the assays themselves, or human error (65).. Furthermore, one notable challenge in our analysis is the partial agonism exhibited by substances like aripiprazole and cariprazine at the 5-HT1A receptor. Partial agonists bind to the receptor and activate it, but to a lesser extent than full agonists, resulting in a mixed pharmacological profile that can influence both therapeutic and adverse effects. This also presents a challenge in accurately gauging the extent to which the substance exerts agonistic or antagonistic effects. Consequently, partial agonists cannot be evaluated as a group as having the same degree of agonistic or antagonistic impact. This limitation is particularly evident when calculating receptor occupancy. While the formula for calculating receptor occupancy is highly suitable, provided that the drug concentration in the blood is provided. However, this can only be roughly estimated in data from spontaneous reporting databases since the concentration is usually unknown. Even when the daily dose in milligrams is known, estimating the concentration at the individual level is challenging due to the significant influence of other factors. In order to ensure comparability with other studies, an estimate based on the upper therapeutic range was also selected in our study. However, it is unlikely that the majority of patients regularly take medication in the recommended dosage at the upper end of the therapeutic range. In light of these considerations, the calculation of occupancy is susceptible to error. To address this, we conducted an additional calculation for the pKi.

In addition while there was a significant association between higher affinities for the 5-HT1A and reduced reporting of DILI, it is not possible to evaluate the temporal link of these events with our study. Another factor that warrants consideration is the issue of drug interaction, which can play an important role in DILI (5, 66). In our exploratory approach, we were unable to sufficiently account for the aspect of simultaneous exposure to different drugs in the sense of drug interaction. This represents a significant challenge in the field of pharmacovigilance analyses in general (67). A promising approach was recently described in a paper by Battini et al. using machine learning, which represents a potential approach for future investigations (68). Another limitation is the investigation of DILI. Pharmacovigilance databases are generally suitable for investigating rare adverse drug reactions. In the case of DILI, however, the problem arises that it is a diagnosis of exclusion. The detailed data required for this is not available to us as part of the analysis, meaning that the SMQ “drug related hepatic disorder” should only be understood as an estimate. One potential approach for addressing this limitation in future studies could be a case-by-case analysis of ICSRs or the utilization of data from a national health register. Finally, as already mentioned, it is essential to bear in mind that disproportionality analysis does not allow causal inference or should be considered equivalent to incidence or relative risk. Consequently, further longitudinal studies are needed to confirm causality and elucidate the underlying biological pathways suggested by our findings.

### 4.4 Conclusion

The focus of this study was a pharmacoepidemiological-pharmacodynamic approach to investigate the potential role of receptor affinity of antipsychotics in DILI in an exploratory manner. Our findings indicate that, despite the acknowledged limitations, a pharmacoepidemiological-pharmacodynamic approach can be employed to identify risk and protective factors, in this case in the use of antipsychotics and the risk of hepatotoxicity. The results are consistent with experimental findings from animal models and models with human cells, suggesting a role for the serotonin system with regard to hepatotoxicity. Our data show initial evidence that substances with an affinity for the serotonin system are associated with a lower occurence of DILI. In clinical practice, this potential effect should be taken into account above all in patients with a schizophrenic disorder who are affected by other risk factors that favour DILI or who are dependent on antipsychotic therapy despite a previously damaged liver. Subsequent studies should investigate the potential role of additional characteristics in the development of DILI, particularly for substances that have generated a signal. These characteristics may include concomitant drugs and adverse events, ethnicity, time to onset, discontinuation and dechallenge, and, if possible, causality assessment, possibly with the help of a case-by-case assessment in sponatenous reporting databases or with large electronical health databases.

## Data Availability

This study analyzed publicly available data sets. The datasets can be found at https://openvigil.sourceforge.net/ and https://www.fda.gov/drugs/questions-and-answers-fdas-adverse-event-reporting-system-faers/fda-adverse-event-reporting-system-faers-public-dashboard.
