# Supplementary material for: Hepatotoxicity of antipsychotics: an exploratory pharmacoepidemiologic and pharmacodynamic study integrating FAERS data and in vitro receptor-binding affinities
Source: Front Psychiatry. 2024 Oct 14;15:1479625. doi: 10.3389/fpsyt.2024.1479625 (PMC11513306; doi:10.3389/fpsyt.2024.1479625)
Supplement: Supplementary file 1 [file Table1.docx]

Supplementary Material

Table S1. Number of cases. Only substances with n ≥ 3 were included in the analysis

| Number of entire cases | | | | |
| --- | --- | --- | --- | --- |
|  | **Total** | **Female** | **Male** | **Age ≥ 65 y** |
| **Substance** |  |  |  |  |
| Aripiprazole | 1489 | 596 | 695 | 125 |
| Asenapine | 66 | 30 | 26 | 2 |
| Brexpiprazole | 54 | 29 | 24 | 7 |
| Cariprazine | 42 | 17 | 12 | 1 |
| Chlorpromazine | 276 | 121 | 125 | 59 |
| Clozapine | 1963 | 662 | 1182 | 126 |
| Fluphenazine | 44 | 17 | 21 | 2 |
| Haloperidol | 467 | 165 | 254 | 82 |
| Iloperidone | 5 | 5 | 0 | 0 |
| Loxapine | 205 | 87 | 100 | 23 |
| Lurasidone | 119 | 48 | 38 | 3 |
| Olanzapine | 2521 | 1055 | 1287 | 307 |
| Paliperidone | 305 | 85 | 173 | 7 |
| Perphenazine | 34 | 23 | 7 | 3 |
| Pimozide | 11 | 5 | 5 | 0 |
| Quetiapine | 2802 | 1531 | 1052 | 411 |
| Risperidone | 1725 | 662 | 854 | 241 |
| Thiothixene | 8 | 4 | 3 | 0 |
| Ziprasidone | 166 | 75 | 64 | 7 |
|  |  |  |  |  |

**Table S2.** Information Component (IC) and the respective 95% confidence interval for drug related hepatic disorders - comprehensive search (SMQ) and the categories “Total”, “Female”, “Male”, and “Age ≥ 65 years”. Signals identified are presented in bold font. A signal is defined as a lower 95% confidence interval greater than 0. NA = no data available or number of individual case safety reports is <3.

| ICs incl. 95 % Confidence Interval | | | | |
| --- | --- | --- | --- | --- |
|  | **Total** | **Female** | **Male** | **Age ≥ 65 y** |
| **Substance** | **IC (95 % CI)** | **IC (95 % CI)** | **IC (95 % CI)** | **IC (95 % CI)** |
| Aripiprazole | -0.39 (-0.49, -0.29) | \| -0.41 (-0.57, -0.26) \| \| --- \| | \| -0.25 (-0.39, -0.10) \| \| --- \| | \| -0.03 (-0.38, 0.33) \| \| --- \| |
| Asenapine | -1.56 (-1.96, -1.16) | \| -1.56 (-2.15, -0.97) \| \| --- \| | \| -1.44 (-2.08, -0.79) \| \| --- \| | \| NA \| \| --- \| |
| Brexpiprazole | -2.27 (-2.69, -1.85) | \| -2.21 (-2.79, -1.64) \| \| --- \| | \| -1.78 (-2.43, -1.14) \| \| --- \| | \| -1.52 (-2.72, -0.32) \| \| --- \| |
| Cariprazine | -1.55 (-2.05, -1.05) | \| -1.63 (-2.40, -0.85) \| \| --- \| | \| -1.72 (-2.63, -0.81) \| \| --- \| | \| NA \| \| --- \| |
| Chlorpromazine | **1.30 (0.98, 1.62)** | \| **1.46 (0.97, 1.96)** \| \| --- \| | \| **1.08 (0.64, 1.53)** \| \| --- \| | \| **1.64 (0.89, 2.38)** \| \| --- \| |
| Clozapine | -0.36 (-0.45, -0.28) | \| -0.16 (-0.32, -0.01) \| \| --- \| | \| -0.42 (-0.53, -0.31) \| \| --- \| | \| -1.32 (-1.62, -1.03) \| \| --- \| |
| Fluphenazine | 0.67 (-0.01, 1.35) | \| 0.99 (-0.17, 2.17) \| \| --- \| | \| 0.24 (-0.66, 1.14) \| \| --- \| | \| NA \| \| --- \| |
| Haloperidol | 0.03 (-0.16, 0.21) | \| 0.11 (-0.21, 0.42) \| \| --- \| | \| -0.08 (-0.33, 0.16) \| \| --- \| | \| 0.14 (-0.31, 0.59) \| \| --- \| |
| Iloperidone | -2.39 (-3.70, -1.07) | \| -1.09 (-2.55, 0.37) \| \| --- \| | \| NA \| \| --- \| | \| NA \| \| --- \| |
| Loxapine | **1.38 (1.00, 1.75)** | \| **1.56 (0.96, 2.16)** \| \| --- \| | \| **1.10 (0.60, 1.60)** \| \| --- \| | \| **1.24 (0.17, 2.31)** \| \| --- \| |
| Lurasidone | -1.86 (-2.15, -1.56) | \| -2.04 (-2.49, -1.58) \| \| --- \| | \| -1.68 (-2.20, -1.16) \| \| --- \| | \| -2.46 (-4.11, -0.82) \| \| --- \| |
| Olanzapine | **0.78 (0.68, 0.87)** | \| **0.93 (0.78, 1.07)** \| \| --- \| | \| **0.64 (0.52, 0.77)** \| \| --- \| | \| **0.59 (0.34, 0.85)** \| \| --- \| |
| Paliperidone | -1.75 (-1.93, -1.56) | \| -1.07 (-1.44, -0.70) \| \| --- \| | \| -1.87 (-2.11, -1.62) \| \| --- \| | \| -1.43 (-2.63, -0.22) \| \| --- \| |
| Perphenazine | 0.29 (-0.43, 1.01) | \| 0.88 (-0.10, 1.86) \| \| --- \| | \| -0.82 (-2.11, 0.47) \| \| --- \| | \| -0.12 (-2.22, 1.97) \| \| --- \| |
| Pimozide | 0.17 (-1.04, 1.39) | \| 0.57 (-1.33, 2.47) \| \| --- \| | \| 0.13 (-1.61, 1.88) \| \| --- \| | \| NA \| \| --- \| |
| Quetiapine | **0.17 (0.09, 0.25)** | \| **0.36 (0.26, 0.47)** \| \| --- \| | \| -0.10 (-0.22, 0.02) \| \| --- \| | \| 0.17 (-0.03, 0.38) \| \| --- \| |
| Risperidone | -0.27 (-0.36, -0.18) | \| **0.56 (0.39, 0.74)** \| \| --- \| | \| -0.73 (-0.85, -0.61) \| \| --- \| | \| **0.31 (0.04, 0.58)** \| \| --- \| |
| Thiothixene | -0.04 (-1.40, 1.33) | \| 0.01 (-1.88, 1.90) \| \| --- \| | \| -0.05 (-2.17, 2.07) \| \| --- \| | \| NA \| \| --- \| |
| Ziprasidone | -0.94 (-1.21, -0.67) | \| -0.90 (-1.31, -0.50) \| \| --- \| | \| -0.98 (-1.41, -0.55) \| \| --- \| | \| -1.13 (-2.37, 0.12) \| \| --- \| |
|  |  |  |  |  |

| **Table S3. Pearson correlation coefficients r along with their corresponding p-values for the overall analysis, as well as separately for female and male.** | | | |
| --- | --- | --- | --- |
|  | **Total** | **Female** | **Male** |
| **Parameter** | **r (p-value)** | **r (p-value)** | **r (p-value)** |
| \| alpha1-receptor affinity \|  \| \| --- \| --- \| | \| -0.223 (0.358) \| \| --- \| | \| -0.190 (0.437) \| \| --- \| | \| -0.145 (0.567) \| \| --- \| |
| \| alpha2-receptor affinity \|  \| \| --- \| --- \| | \| -0.375 (0.114) \| \| --- \| | \| -0.383 (0.105) \| \| --- \| | \| -0.361 (0.141) \| \| --- \| |
| \| D2-receptor affinity \|  \| \| --- \| --- \| | \| -0.234 (0.334) \| \| --- \| | \| -0.206 (0.397) \| \| --- \| | \| -0.301 (0.224) \| \| --- \| |
| \| D3-receptor affinity \|  \| \| --- \| --- \| | \| -0.112 (0.649) \| \| --- \| | \| -0.059 (0.809) \| \| --- \| | \| -0.207 (0.409) \| \| --- \| |
| \| H1-receptor affinity \|  \| \| --- \| --- \| | \| 0.149 (0.543) \| \| --- \| | \| 0.139 (0.570) \| \| --- \| | \| 0.137 (0.589) \| \| --- \| |
| \| 5-HT1A-receptor affinity \|  \| \| --- \| --- \| | \| -0.684 (0.001) \| \| --- \| | \| -0.735 (0.0003) \| \| --- \| | \| -0.646 (0.004) \| \| --- \| |
| \| 5-HT2A-receptor affinity \|  \| \| --- \| --- \| | \| -0.296 (0.219) \| \| --- \| | \| -0.242 (0.317) \| \| --- \| | \| -0.238 (0.341) \| \| --- \| |
| \| 5-HT2C-receptor affinity \|  \| \| --- \| --- \| | \| -0.089 (0.716) \| \| --- \| | \| -0.116 (0.636) \| \| --- \| | \| -0.057 (0.821) \| \| --- \| |
| \| 5-HT7-receptor affinity \|  \| \| --- \| --- \| | \| -0.375 (0.114) \| \| --- \| | \| -0.329 (0.169) \| \| --- \| | \| -0.436 (0.071) \| \| --- \| |
| \| Muscarinergic-receptor affinity \|  \| \| --- \| --- \| | \| 0.458 (0.048) \| \| --- \| | \| 0.400 (0.090) \| \| --- \| | \| 0.470 (0.049) \| \| --- \| |
| \| Occupancy_alpha1 \|  \| \| --- \| --- \| | \| -0.160 (0.512) \| \| --- \| | \| -0.139 (0.571) \| \| --- \| | \| -0.077 (0.760) \| \| --- \| |
| \| Occupancy_alpha2 \|  \| \| --- \| --- \| | \| -0.213 (0.381) \| \| --- \| | \| -0.187 (0.444) \| \| --- \| | \| -0.249 (0.319) \| \| --- \| |
| \| Occupancy_D2 \|  \| \| --- \| --- \| | \| -0.163 (0.504) \| \| --- \| | \| -0.192 (0.430) \| \| --- \| | \| -0.236 (0.346) \| \| --- \| |
| \| Occupancy_D3 \|  \| \| --- \| --- \| | \| -0.004 (0.987) \| \| --- \| | \| 0.022 (0.928) \| \| --- \| | \| -0.120 (0.636) \| \| --- \| |
| \| Occupancy_H1 \|  \| \| --- \| --- \| | \| 0.160 (0.512) \| \| --- \| | \| 0.122 (0.619) \| \| --- \| | \| 0.166 (0.509) \| \| --- \| |
| \| Occupancy_5-HT1A \|  \| \| --- \| --- \| | \| -0.449 (0.054) \| \| --- \| | \| -0.526 (0.021) \| \| --- \| | \| -0.449 (0.062) \| \| --- \| |
| \| Occupancy_5-HT2A \|  \| \| --- \| --- \| | \| -0.227 (0.351) \| \| --- \| | \| -0.232 (0.339) \| \| --- \| | \| -0.123 (0.626) \| \| --- \| |
| \| Occupancy_5-HT2C \|  \| \| --- \| --- \| | \| 0.053 (0.830) \| \| --- \| | \| 0.008 (0.974) \| \| --- \| | \| 0.065 (0.797) \| \| --- \| |
| \| Occupancy_5-HT7 \|  \| \| --- \| --- \| | \| -0.286 (0.235) \| \| --- \| | \| -0.273 (0.258) \| \| --- \| | \| -0.325 (0.188) \| \| --- \| |
| \| Occupancy_Muscarinic \|  \| \| --- \| --- \| | \| 0.337 (0.158) \| \| --- \| | \| 0.279 (0.247) \| \| --- \| | \| 0.376 (0.124) \| \| --- \| |
| \| MW \|  \| \| --- \| --- \| | \| -0.474 (0.040) \| \| --- \| | \| -0.413 (0.078) \| \| --- \| | \| -0.471 (0.048) \| \| --- \| |
| \| LogP \|  \| \| --- \| --- \| | \| -0.099 (0.685) \| \| --- \| | \| -0.123 (0.616) \| \| --- \| | \| -0.034 (0.895) \| \| --- \| |
| \| Year of Approval \| \| --- \| | \| -0.499 (0.029) \| \| --- \| | \| -0.487 (0.034) \| \| --- \| | \| -0.487 (0.041) \| \| --- \| |

**Table S4: pKi-values for the receptors investigated.**

| Substance | alpha1 | alpha2 | D2 | D3 | H1 | 5-HT1A | 5-HT2A | 5-HT2C | 5-HT7 | muscarinic |
| --- | --- | --- | --- | --- | --- | --- | --- | --- | --- | --- |
| Aripiprazole | 7.52 | 7.13 | 9.08 | 8.89 | 7.54 | 8.35 | 8.06 | 7.65 | 7.99 | 5.45 |
| Asenapine | 8.93 | 9.10 | 8.49 | 8.03 | 9.80 | 8.03 | 10.22 | 10.52 | 9.96 | 4.82 |
| Brexpiprazole | 9.77 | 9.23 | 9.52 | 8.96 | 7.72 | 9.92 | 9.33 | 7.47 | 8.43 | 6.00 |
| Cariprazine | 6.81 | 6.00 | 9.23 | 10.07 | 7.64 | 8.52 | 7.72 | 6.87 | 6.95 | 6.00 |
| Chlorpromazine | 8.95 | 7.11 | 8.40 | 8.52 | 8.29 | 5.51 | 8.56 | 7.81 | 7.45 | 7.15 |
| Clozapine | 8.17 | 7.47 | 6.84 | 6.61 | 8.74 | 6.85 | 8.19 | 7.87 | 7.40 | 7.98 |
| Fluphenazine | 8.04 | 6.50 | 9.24 | 9.52 | 7.68 | 6.84 | 7.94 | 6.01 | 8.10 | 5.72 |
| Haloperidol | 8.07 | 6.22 | 8.72 | 8.40 | 5.92 | 5.72 | 7.10 | 5.33 | 6.34 | 5.00 |
| Iloperidone | 9.51 | 6.79 | 8.01 | 7.98 | 7.91 | 7.03 | 9.80 | 7.37 | 6.95 | 5.26 |
| Loxapine | 7.51 | 6.82 | 7.70 | 7.67 | 8.30 | 5.59 | 8.60 | 7.88 | 7.06 | 6.34 |
| Lurasidone | 7.32 | 7.59 | 8.77 | 7.80 | 6.00 | 8.17 | 8.69 | 6.38 | 9.31 | 6.00 |
| Olanzapine | 7.15 | 6.73 | 7.59 | 7.35 | 8.62 | 5.69 | 8.49 | 7.99 | 6.64 | 7.66 |
| Paliperidone | 8.60 | 7.96 | 8.46 | 9.05 | 8.25 | 6.22 | 8.96 | 7.32 | 8.57 | 5.06 |
| Perphenazine | 8.00 | 6.36 | 9.04 | 9.64 | 8.10 | 5.92 | 8.25 | 6.88 | 7.64 | 5.82 |
| Pimozide | 6.86 | 6.09 | 8.82 | 9.41 | 6.45 | 6.28 | 7.55 | 6.06 | 9.30 | 6.10 |
| Quetiapine | 7.68 | 6.13 | 6.31 | 6.47 | 8.06 | 6.51 | 6.70 | 5.85 | 6.51 | 5.85 |
| Risperidone | 8.30 | 8.02 | 8.57 | 8.09 | 7.72 | 6.38 | 9.54 | 7.60 | 8.37 | 5.00 |
| Thiothixene | 7.92 | 7.28 | 9.38 | 9.40 | 8.36 | 6.39 | 7.30 | 5.87 | 7.82 | 5.00 |
| Ziprasidone | 7.89 | 6.89 | 8.39 | 8.14 | 7.35 | 8.49 | 9.41 | 9.09 | 8.18 | 5.61 |

**Table S5: Occupancy-values (%) for the receptors investigated**

| Substance | alpha1 | alpha2 | D2 | D3 | H1 | 5-HT1A | 5-HT2A | 5-HT2C | 5-HT7 | muscarinic |
| --- | --- | --- | --- | --- | --- | --- | --- | --- | --- | --- |
| Aripiprazole | 20.58 | 9.57 | 90.36 | 85.76 | 21.25 | 63.60 | 47.36 | 25.89 | 43.18 | 0.22 |
| Asenapine | 42.84 | 52.61 | 21.20 | 8.59 | 84.57 | 8.59 | 93.60 | 96.69 | 88.85 | 0.01 |
| Brexpiprazole | 95.00 | 84.56 | 91.51 | 74.61 | 14.54 | 96.42 | 87.30 | 8.68 | 46.62 | 0.32 |
| Cariprazine | 1.78 | 0.28 | 82.68 | 97.07 | 10.91 | 48.41 | 12.91 | 2.06 | 2.47 | 0.28 |
| Chlorpromazine | 98.82 | 54.73 | 95.93 | 96.92 | 94.85 | 2.99 | 97.14 | 85.85 | 72.66 | 57.15 |
| Clozapine | 89.03 | 61.88 | 27.78 | 18.46 | 96.82 | 28.28 | 89.45 | 80.36 | 57.98 | 84.02 |
| Fluphenazine | 9.19 | 0.29 | 61.62 | 75.31 | 4.18 | 0.62 | 7.32 | 0.09 | 10.26 | 0.05 |
| Haloperidol | 22.92 | 0.42 | 57.26 | 39.01 | 0.21 | 0.13 | 3.07 | 0.05 | 0.56 | 0.03 |
| Iloperidone | 79.10 | 0.72 | 10.69 | 10.01 | 8.71 | 1.24 | 88.00 | 2.67 | 1.04 | 0.02 |
| Loxapine | 2.87 | 0.60 | 4.38 | 4.09 | 15.50 | 0.04 | 26.84 | 6.47 | 1.04 | 0.20 |
| Lurasidone | 4.84 | 8.65 | 59.20 | 13.44 | 0.24 | 26.53 | 54.56 | 0.58 | 83.12 | 0.24 |
| Olanzapine | 20.40 | 8.80 | 41.16 | 28.66 | 88.30 | 0.86 | 84.59 | 63.68 | 7.32 | 45.14 |
| Paliperidone | 93.61 | 76.89 | 91.39 | 97.60 | 86.71 | 5.70 | 97.07 | 43.26 | 93.13 | 0.41 |
| Perphenazine | 4.00 | 0.09 | 31.17 | 64.44 | 4.95 | 0.03 | 6.93 | 0.31 | 1.78 | 0.03 |
| Pimozide | 0.31 | 0.05 | 22.14 | 52.65 | 0.12 | 0.08 | 1.52 | 0.05 | 46.42 | 0.05 |
| Quetiapine | 91.39 | 22.90 | 30.95 | 39.48 | 96.23 | 41.79 | 52.59 | 13.68 | 41.79 | 13.68 |
| Risperidone | 83.26 | 72.25 | 90.21 | 75.37 | 56.69 | 5.61 | 98.85 | 49.86 | 85.26 | 0.25 |
| Thiothixene | 16.49 | 4.36 | 85.09 | 85.56 | 35.00 | 0.57 | 4.52 | 0.17 | 13.45 | 0.02 |
| Ziprasidone | 27.18 | 3.63 | 54.21 | 40.03 | 9.76 | 59.85 | 92.56 | 85.67 | 42.32 | 0.20 |

**Table S6: Molecular Weight, Lipophilicity and Year of Approval**

| Substance | MW (g/mol) | LogP | Year of Approval |
| --- | --- | --- | --- |
| Aripiprazole | 447.15 | 5.21 | 2002 |
| Asenapine | 285.09 | 4.35 | 2009 |
| Brexpiprazole | 433.18 | 5.38 | 2015 |
| Cariprazine | 426.2 | 4.56 | 2015 |
| Chlorpromazine | 318.1 | 5.18 | 1954 |
| Clozapine | 326.13 | 3.67 | 1989 |
| Fluphenazine | 437.17 | 4.4 | 1967 |
| Haloperidol | 375.14 | 3.7 | 1967 |
| Iloperidone | 426.2 | 4.26 | 2009 |
| Loxapine | 327.11 | 3.18 | 2012 |
| Lurasidone | 492.26 | 5.25 | 2010 |
| Olanzapine | 312.14 | 3.61 | 1996 |
| Paliperidone | 426.21 | 2.3 | 2006 |
| Perphenazine | 403.15 | 4.15 | 1998 |
| Pimozide | 461.23 | 6.36 | 1985 |
| Quetiapine | 383.17 | 2.93 | 1997 |
| Risperidone | 410.21 | 3.27 | 1993 |
| Thiothixene | 443.17 | 4.01 | 1967 |
| Ziprasidone | 412.11 | 4.64 | 2001 |
